# Evaluation of Uliginosin B as a Modulator of the Ras Pathway: In Vivo Evidence From *Caenorhabditis elegans* and In Silico Insights

**DOI:** 10.1002/cbdv.71592

**Published:** 2026-08-17

**Authors:** Maria Eduarda O. De Souza, Flávia Suelen De O. Pereira, Daniela Teixeira Rodrigues, Helder Dias Costa, Tarsila Dantas Da Rosa, Camila Machado Pires da Silva, Gilsane von Poser, Stela M. K. Rates, Favero R. Paula, Daiana Silva Ávila

**Affiliations:** ^1^ Research Group on Biochemistry and Toxicology in Caenorhabditis elegans Graduation Program in Biochemistry Federal University of Pampa Uruguaiana Rio Grande do Sul Brazil; ^2^ Northwest Regional University of the State of Rio Grande Do Sul Ijuí Rio Grande do Sul Brazil; ^3^ Graduation Program in Biological Sciences ‐ Toxicological Biochemistry Federal University of Santa Maria Rio Grande do Sul Brazil; ^4^ Laboratory of Research and Drugs Development Graduation Program in Pharmaceutical Sciences Federal University of Pampa Uruguaiana Rio Grande do Sul Brazil; ^5^ Integrated Regional University of Alto Uruguai and Missões Frederico Westphalen Rio Grande do Sul Brazil; ^6^ Graduation Program in Pharmaceutical Sciences Federal University of Rio Grande Do Sul Porto Alegre Rio Grande do Sul Brazil

**Keywords:** acylphloroglucinol, alternative model, cancer, molecular docking, natural compounds

## Abstract

Previously, in vitro studies have suggested a possible antiproliferative action of uliginosin B (ULI B), a dimeric acylphloroglucinol isolated from *Hypericum* species. However, no in vivo evidence of this effect has been found yet. Therefore, the aim is to evaluate its possible interaction in the Ras pathway using the nematode *Caenorhabditis elegans* and computational models. For the in vivo assays, we used a strain with a gain‐of‐function in the *let‐60* gene, which is a human Ras homologous protein that leads to the formation of tumorous multivulva. The experimental tests indicated that a single 30 min treatment with ULI B was safe regarding toxicological parameters, and the worms demonstrated a delay in the multivulva phenotype development (MV) on day 1 and 2 of adulthood, with statistical significance at 5, 10, and 20 µM. In silico, ULI B showed a possible allosteric and non‐competitive interaction with LET‐60, but not as significant as in the human Ras, possibly justifying the weak effect found with the delay in MV development in the nematode. However, based on the in‐silico data, ULI B seems promising against human cancer cells, as its interactions with EGFR and HRAS were more favorable than those with *C. elegans* proteins.

## Graphical abstract

This study performs in vivo and in silico assays with uliginosin B, targeting its activity in the Ras signaling pathway, demonstrating a possible allosteric interaction with the HRAS (human) and LET‐60 (nematode) proteins, and a decrease in the overexpression of the *let‐60* gene in the nematode *C. elegans*.

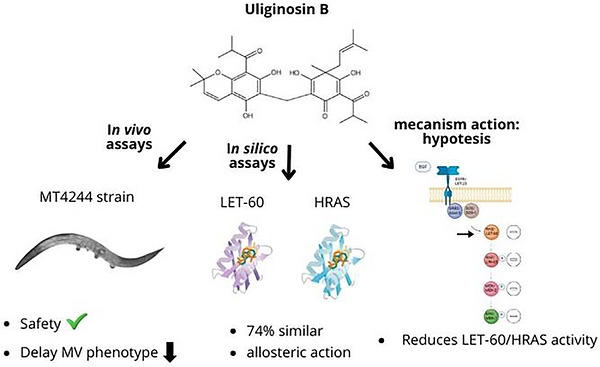

## 1 Introduction

An oncogenic condition is presented by abnormal cellular aspects that manifest due to changes in gene expression, originating from endogenous or exogenous (environmental) mutagenic agents [1]. According to the International Agency for Research on Cancer (IARC), an increase of around 19.3 million cases and 10 million deaths has been reported, since the efficacy of the therapies is not often successful [2]. These data contribute to the relevance of the search for new drugs with more effective antitumor action and fewer adverse effects [3]. In a study by Kang and colleagues, phloroglucinol, a natural phlorotannin, was used to investigate its anticarcinogenic effect on the insulin‐like growth factor‐1 receptor (IGF‐1R) in HT‐29 human colon cancer cells [4]. The treatment significantly inhibited the expression of Ras, Raf, and the mammalian target of rapamycin (mTOR), consequently reducing the viability of colon cancer cells. Anticancer medicines that originated from natural compounds account for around 60% of all chemotherapies, and in the last 30 years, there has been a significant increase in studies with natural products with cytotoxic action that have contributed to the development of the current therapies. Therefore, the importance of ensuring the efficacy and safety of these molecules and the search for new therapeutic sources attracts attention [5].

**FIGURE 1:**
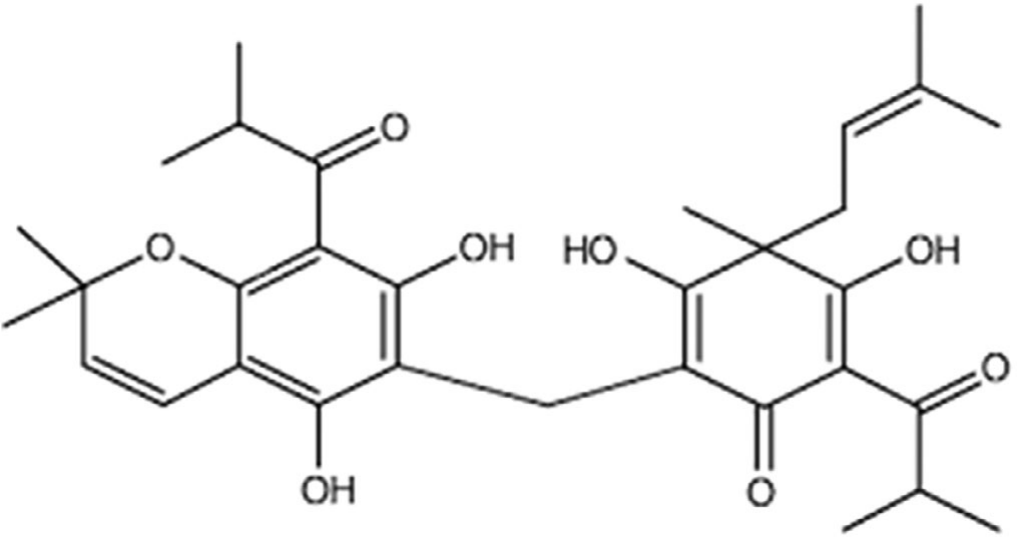
Chemical structure of ULI B (Sakamoto et al., 2012).

Phloroglucinol derivatives are an important class of secondary metabolites, included within the class of phenolic compounds. Among them, uliginosin B (ULI B) is present in different species of *Hypericum* (Figure 1). Previous studies have already demonstrated the antidepressant‐like effect of ULI B, which could be related to its ability to inhibit the synaptosomal uptake of monoamines and increase the activity of the brain Na+K+‐ATPase enzyme [6, 7]. Its antinociceptive effect seems to be linked to monoaminergic and glutamatergic neurotransmission, adenosine accumulation in brain tissue, and activation of the opioid system, directly affecting the inhibition of NTPDase enzymes [8, 9, 10]. In addition, it presented an anticonvulsant effect [11]. It has been observed that its mechanism may also be involved with the increase in hippocampal reduced glutathione (GSH), monocyte chemotactic protein‐1 (MCP‐1), and interleukin‐10 (IL‐10) [12]. Other effects have been reported in the literature, including antimicrobial and antibiofilm action [13, 14], anti‐*Trichomonas vaginalis* [15], effects against *Leishmania amazonensis* [16], proliferation of healing cells [17], and potentiation of analgesic action through modulation of the adenosinergic system [18, 19]. Notably, a possible antiproliferative action in the OVCAR‐3 cell line has been reported, which has been shown to be promising for the development of new drugs and highest druggability considering Lipinski's rule of five [20, 21, 22].

The Ras signaling pathway is one of the heavily researched targets for the development of cancer therapies. This pathway is responsible for cell proliferation and differentiation, cell cycle, and anti‐apoptosis action [23]. The *Ras* gene, when deregulated by mutations in its isoform, is related to almost a third of all types of human cancers, such as endocrine glands and the gastrointestinal system [24]. Remarkably, the nematode *Caenorhabditis elegans* presents a homologous gene to Ras, named *let‐60*. A genetic analysis showed that the nematode gene encodes a protein structure with 84% similarity, with 164 conserved amino acids of the protein encoded by the Ras gene from other species, thus maintaining its biochemical functions [25]. Due to the availability of transgenic tumorous animals, it is already a well‐established model for screening anti‐tumoral candidates [26].

In *C. elegans, the* Ras pathway triggers the expression of genes that control oocyte formation, progression of the meiotic cycle, specification of sperm fate, spermatogenesis, vulva development, and apoptosis [27]. The MT4244 transgenic strain presents a gain‐of‐function of the *let‐60* gene, which belongs to a pathway signaled by the interaction of its ligand (for example, EGF) with the LET‐23 receptor (homologous to the human EGFR). Therefore, it has a positive regulation in the vulva formation, leading to the development of the multivulva phenotype, which is tumorous [28]. Because of the proliferation control, the Ras pathway is a great target for chemotherapy agents, particularly for initial screening in *C. elegans* [29, 30].

Notably, the free‐living nematode *C. elegans* has around 60%–80% of genes orthologous to humans, and for this reason, they have facilitated the development of cellular markers for gene or protein expression alterations [31]. Furthermore, the laboratory cultivation is inexpensive, as it is a hermaphrodite worm, with a maximum length of 1 mm when adult, a transparent body, a short reproductive cycle, and a lifespan. Among these advantages, it becomes a model that facilitates the understanding of biochemical pathways related to human diseases in pharmacology and for the extrapolation of some findings to humans [32].

Previous studies have demonstrated that ULI B exhibits effects against tumor cell lines, which motivated us to conduct this investigation. In addition, it was recently observed that ULI B has toxicity in wild‐type *C. elegans* at high concentrations, which is essential for its anti‐hyperplastic action [33]. Still, little is known about the antiproliferative mechanism of ULI B, therefore, this research seeks to evaluate its safety and effect as an antitumoral drug for the first time in an *in vivo* model, using a strain with hyperplasia in *C. elegans*, in addition to molecular modeling tools to understand the involvement of the Ras pathway [34, 35].

Therefore, the antiproliferative effects of ULI B, a plant‐derived molecule, through the RAS pathway in in vivo models are still unknown. Hence, for the first time, the action of ULI B was evaluated in a model of hyperplasia in the nematode *C. elegans* caused by overexpression of the *let‐60* gene, which is homologous to the human *hras* gene. Furthermore, we used bioinformatics to elucidate possible mechanisms by which ULI B can interact with the LET‐60/RAS pathway.

## 2 Materials and Methods

### 2.1 Chemicals

Dimethyl sulfoxide (DMSO) PA was obtained from Synth (Diadema, SP, Brazil). Hydrochloric acid (HCl), ethanol, ammonium hydroxide (NH4OH), and sodium hydroxide (NaOH) were acquired from Labsynth (Diadema, SP, Brazil). For the *C. elegans* analysis, we use levamisole (RIPERCOL L 150F; SP, Brazil). All other reagents were of analytical grade and were obtained from local suppliers.

### 2.2 Extraction and Isolation of ULI B

The aerial parts of *H. polyanthemum* were used to isolate ULI B, which was chemically characterized by chromatographic and spectroscopic methods (STOLZ, 2012) by the research group of Prof. Dr. Gilsane von Poser (Pharmacognosy Laboratory, Faculty of Pharmacy, Universidade Federal do Rio Grande do Sul—UFRGS). The plant material was collected in Caçapava do Sul, RS, in October, 2018. The species was identified by Dr. Sergio Bordignon (UNILASALLE, Canoas, RS, Brazil). Voucher was deposited at the ICN herbarium, UFRGS (3118).

### 2.3 *C. elegans* Strains, Culture Conditions, and Synchronization

The strains used in this study were N2 (wild type) and MT4244 *[unc‐24(e138) let‐60(n1046) IV]* (multivulva phenotype). The animals were kept in the incubator at 20°C and cultivated in the nematode growth medium (NGM), seeded with the bacteria *Escherichia coli OP50* (*E. coli*) as a food source (Stiernagle, 2006). For synchronization, the lysis solution (1 M NaOH, 1% NaClO and H2Od) was used to break the cuticle of gravid adults and to release the eggs. After 14–16 h, the first larval stage (L1) worms hatched and were used for the assays. All nematode strains and *E. coli* were purchased from the *Caenorhabditis* Genetic Center (Minnesota, USA).

### 2.4 Exposure Protocol

For ULI B exposure, 1500 animals at the first larval stage (L1) were used for each treatment group. After hatching, the larvae were divided into a control group, exposed to the vehicle dimethylsulfoxide (DMSO) 5% or treated with ULI B at concentrations of 1, 5, 10, and 20 µM, chosen according to the concentration curve previously carried out [33]. After exposure to ULI B in the liquid medium for 30 min, worms were washed three times with M9 buffer to remove the treatment (characterizing acute exposure). The worms were transferred to Petri dishes with nematode growth medium (NGM) and *E. coli OP50* seeded the day before. The worms were kept in the incubator for 48 h for posterior analysis.

### 2.5 Survival Rate

In this assay, the safety of the compounds for strain MT4244 was evaluated by counting the live worms in each exposed or control group. The scoring was performed 48 h after exposure, in duplicates and individually repeated four times. A grid was used to assist in counting the live worms, and the data was normalized to the percentage of control (vehicle).

### 2.6 Body Length and Body Area

As one of the toxicity parameters, the size of the worms was also measured, both in length and in its body area, 48 h after treatment. The plates containing the treated animals were washed with M9 buffer solution and transferred to microtubes. These were centrifuged and washed three times with M9 buffer and transferred to slides. Images of 10 worms per group were taken in the scale microscope (Nikon eclipse 50i microscope). This experiment was repeated four times.

### 2.7 Brood Size

Worms of the strains N2 and MT4244 at the L4 larval stage were individually transferred to new Petri dishes (30 mm) with *E. coli* OP50 inoculated on the same day. The animals (P0) were transferred daily and monitored for 4 days by scoring the number of larvae (progeny). The assays were performed in triplicate for each group and repeated four times.

### 2.8 Egg Laying and Egg Production Tests

Strains N2 and MT4244 were evaluated in these assays. For the egg‐laying test, two treated worms from each group were transferred to Petri dishes with NGM medium containing 10 µL of levamisole solution (500 mM), a cholinergic agonist that induces the expulsion of the eggs, and another with bacteria *E.coli OP50*. After 1 h, the number of released eggs was counted. The number of eggs of the two animals was averaged. In the egg production test, five worms from each group were individually transferred into five drops of lysis solution (1 M NaOH, 1% NaClO, and distilled H_2_O) on a slide. After 2 min, the cuticle of the animals breaks, and the number of eggs is counted. The number of eggs of the five animals was averaged. Both assays were performed on the first (first) and third (third) day of adult worms. The assays were repeated four times.

### 2.9 Longevity and Phenotype Quantification of Multivulvas

Twenty‐five worms (N2 and MT4244) were transferred to new Petri dishes (30 mm) containing *E. coli OP50*, and kept at 20°C. In the following days, the number of live worms was counted daily until there were no live worms left. The quantification (number of animals that developed MV) and number of MV in each animal was evaluated on days 1, 2, 3, and 5 of MT4244 adults (*n* = 10–15 worms). The percentage of worms with the phenotype (MV) was calculated, as well as the average number of MV for each worm. The experiments were carried out in duplicates and repeated four times.

### 2.10 Quantification of the Area of the MV Phenotype

MT4244 treated worms were used to quantify the area of the MV in five worms from each group, which were chosen at random for imaging. First, from the treatment plates, the worms were transferred to microtubes with M9 buffer solution, washed three times on their first or second day of adulthood to separate the larvae, and then poured onto plates with NGM and *E. coli OP50*. On the following day, second‐ or third‐day adults were washed and transferred to slides along with 20 µL of Levamisole solution to paralyze them and capture images with the microscope (Floid Cell Imaging Station). The quantification of the area of the MV was performed using the Image J software, with the average of the values of the MV of each worm and the average of the area of five (5) worms in each group used for the analysis. The assay was repeated independently 4 times.

### 2.11 In Silico Analysis

#### 2.11.1 Molecular Modeling and Ligand Preparation

The ULI B ligand structure was downloaded from the PubChem database, in pdb format (Protein Data Bank), and was prepared using Spartan’14 software. To calculate the geometry of the molecule, the molecular mechanisms MMFF (Molecular Force Field) followed by the semi‐empirical model AM1 and DFT 6.31G* were chosen, leaving its charge neutral. This structure was transformed into Mol2 format and was used for the docking studies.

The molecular structure of the human GTPase proteins HRAS (PDB code: 8ELZ) and the tyrosine kinase domain of the activated (PDB code: 7KXZ) and inactivated (PDB code: 1M14) endothelial growth factor receptor (EGFR), as well as the homologous proteins in C. elegans, LET‐23 kinase (PDB code: 5WNO) and LET‐60 (AlphaFold: P22981) were obtained from the protein database: PDB (https://www.rcsb.org/search), PDBe (https://www.ebi.ac.uk/pdbe/pdbe‐kb/) and AlphaFold (https://alphafold.ebi.ac.uk/).

Docking studies were performed using two methods: blind and semiflexible. Firstly, blind molecular docking was carried out through the SwissDock web server http://www.swissdock.ch/docking (accessed July 2023 and September 2025). Flexible docking was performed using iGEMDOCK program, programmed to perform Stable Docking (slow), with application of genetic algorithm (300 population size, 80 generations, and 10 number of solutions), and set to scoring function of hydrophobic/electrostatic 1:1, and ligand intra energy activated. For the protein configurations, the reference ligand was retained or not, and using 6 Å for the binding site radius. The validation of molecular docking was carried out by applying redocking with LET‐23 kinase and active EGFR.

#### 2.11.2 Sequence Alignments

The alignment was performed with the amino acid sequences available from GenBank (GTPase protein HRAS isoform 1 – *Homo sapiens*: NP_001123914.1, EGFR—*Homo sapiens*: NP_001333826.1, LET‐23 – *C. elegans*: CAA93882.3 and LET‐60 – *C. elegans*: NP_502213.3) and from Protein Data Bank (LET‐60 ‐ AlphaFold: P22981 and LET‐23 ‐ PDB code: 5WNO). The pairwise MUSCLE alignment, the multiple sequence alignment and annotations were performed using the Unipro UGENE software Version 53.

### 2.12 Statistical Analysis

All assays were repeated at least four times, independently, using different batches of worms. The Shapiro‐Wilk normality test was performed, and when they presented normal distribution (all *p*s < 0.05), the one‐way analysis (ANOVA) was performed, followed by Dunnett's multiple comparison test. When the data did not show normal distribution, the non‐parametric test chosen was the Kruskal–Wallis followed by Dunn's multiple comparisons test. The longevity data were analyzed by Kaplan–Meier. Data were expressed as mean ± standard error of the mean (S.E.M). Results were determined to be significant when the significance was *p < 0.05, **p < 0.01, ***p< 0.001 and ****p < 0.0001, using Graph Pad Prism 8.0.1 software for such analyses.

## 3 Results

### 3.1 Exposure to ULI B did not Alter the Survival Rate and Development of the Animals

**FIGURE 2:**
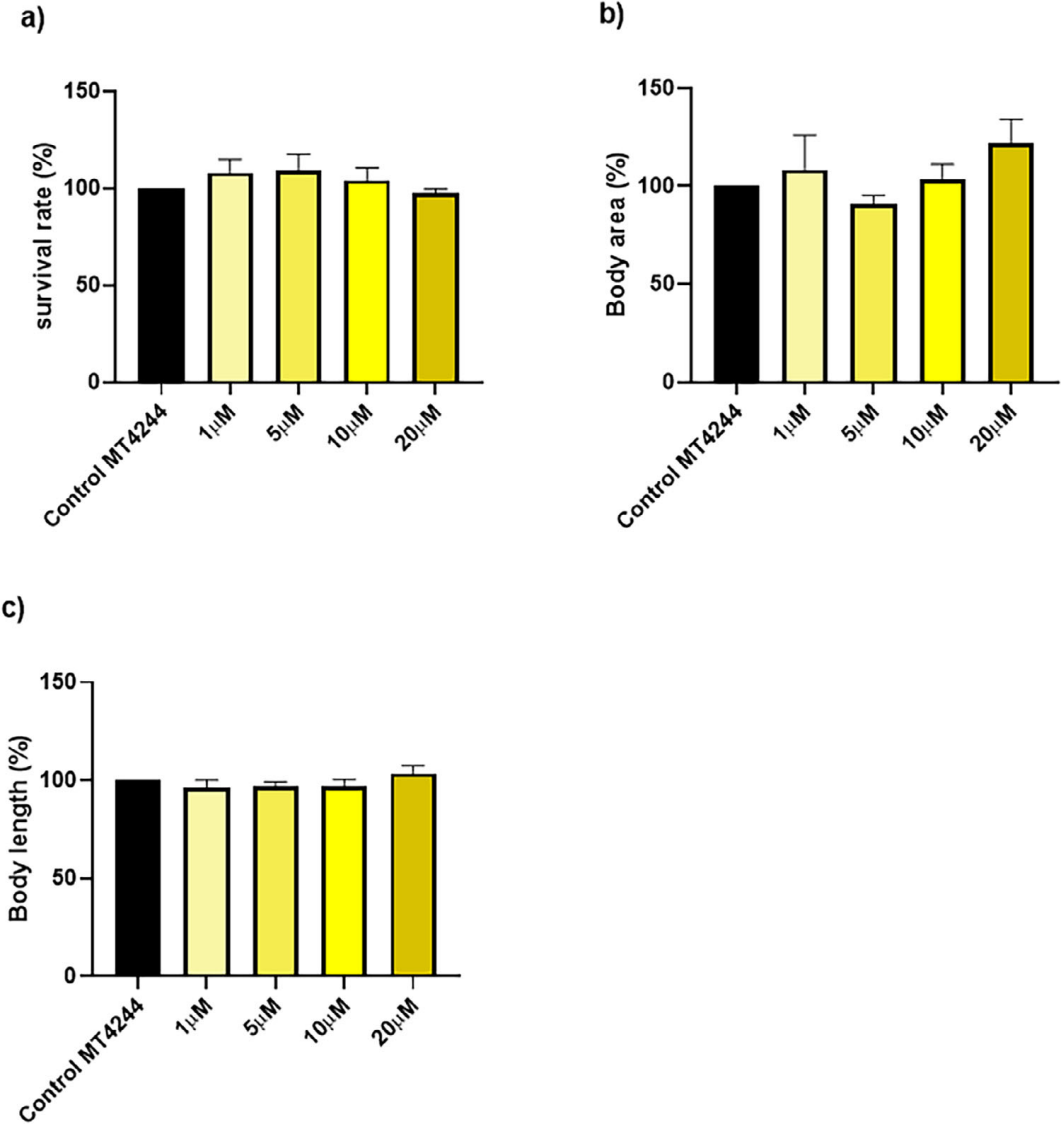
Survival and body size of the MT4244 animals exposed to ULI. (a) Survival rate (*n* = 4). (a) Data were non‐parametric and Kruskal–Wallis followed by Dunn's multiple comparisons test was applied; (b) Body area and (c) Body length (*n* = 4). (b and c) Data were parametric and ANOVA was performed, followed by Dunnett's multiple comparison test. Data were normalized to 100% of control and expressed as mean ± SEM. (*) Indicates a statistically significant difference compared the N2 control group with *p < 0.05, **p < 0.01, ***p < 0.001, and ****p < 0.0001.

In the parameters used to evaluate the compound's toxicity in the nematode (survival and body size), ULI B was safe at the concentrations tested for the MT4244 strain, as shown in Figure 2. It was observed for the development parameters (body area and length) at values similar to the control group (Figure 2b,c).

### 3.2 Exposure to ULI B did not Improve the Reproductive Parameters of the MT4244 Strain

**FIGURE 3:**
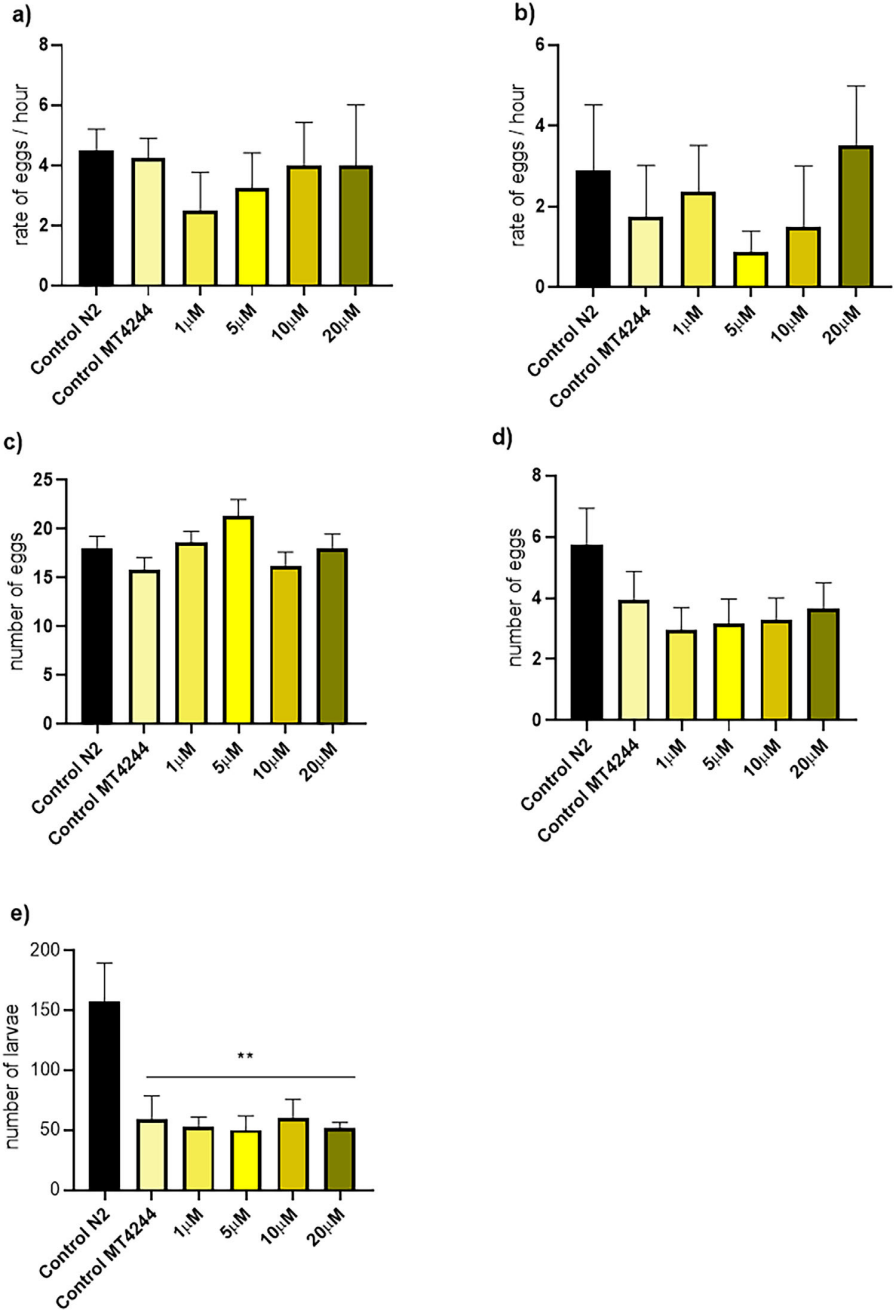
Reproductive parameters in MT4244 worms treated with ULI B. (a) Quantification of the number of eggs expelled without and with the stimulus of levamisole in the worms, on the first and (b) third day of adulthood (*n* = 4). (c) Quantification of the average number of eggs inside the animals, on the first and (d) third day of adulthood (*n* = 4). (e) Size of the progeny of the animals (*n* = 4). (a and e) Data were parametric and ANOVA was performed, followed by Dunnett's multiple comparison test. (b, c, and d) Data were non‐parametric and Kruskal–Wallis followed by Dunn's multiple comparisons test was applied. Data expressed as mean ± SEM. (*) Indicates a statistically significant difference compared the N2 control group with *p < 0.05, **p < 0.01, ***p < 0.001, and ****p < 0.0001.

Regarding the reproductive parameters, the treated worms did not show changes in egg laying (Figure 3a,b), and in the egg production test (Figure 3c,d), both at the first (72 h after exposure) and at the third (120 h after exposure) adult day. In the progeny number assay, it can be observed that ULI B was not able to reverse the progeny reduction phenotype presented by the transgenic strain. Therefore, there was only a significant difference between the strains of the control groups N2 and MT4244 (Figure 3e,f).

### 3.3 Exposure to ULI B did not Extend the Lifespan of the Worms, but Delayed the Development of the Multi‐Vulva Phenotype of MT4244 Animals

**FIGURE 4:**
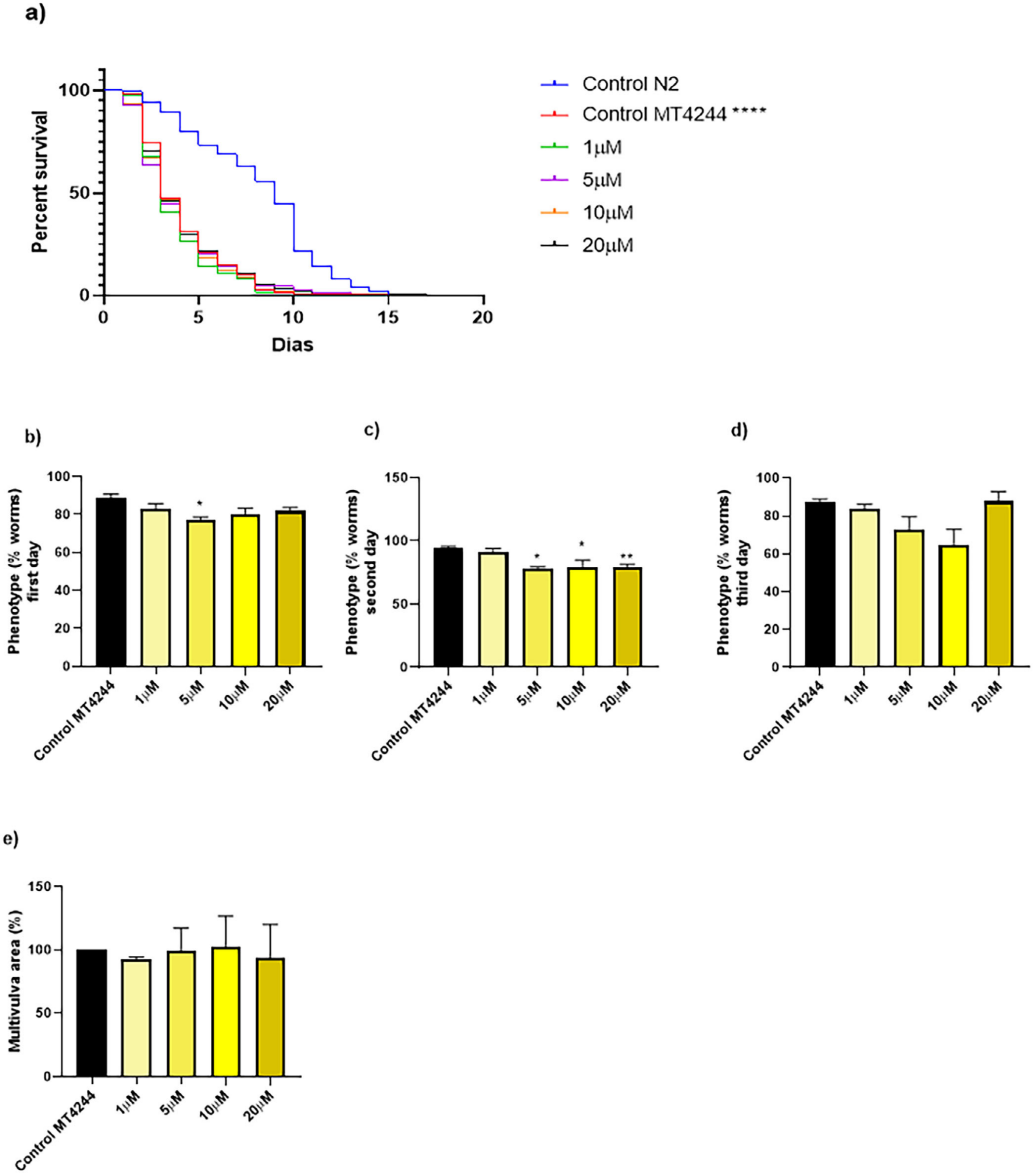
Longevity assay and quantification of animals that developed the MV phenotype after exposure to ULI B. (a) Survival rate over the days (*n* = 4). (b–d) Percentage of worms that developed the phenotype, on the first (b), second (c) third, and (d) day of adulthood (n = 4). (e) Determination of the average MV area of the worms on the third day of adulthood (n = 3). (a) Data were analyzed by Kaplan–Meier. (b, c, e) Data were parametric and ANOVA was performed, followed by Dunnett's multiple comparison test. (d) Data were non‐parametric and Kruskal–Wallis followed by Dunn's multiple comparisons test. (e) Data were normalized to 100% of the control. Data expressed as mean ± SEM. (*) Indicates a statistically significant difference in relation to the control group with *p < 0.05, **p < 0.01, ***p < 0.001, and ****p < 0.0001.

It was possible to observe a significant reduction in the average lifespan of the untreated MT4244 strain compared to the N2 strain, indicating the shorter lifespan of the mutants caused by *let‐60 gf*. However, ULI B did not prolong the lifespan of the mutant strain (Figure 4a). Along with the longevity assay, the quantification of the MV number was carried out. A delay in the formation of these phenotypes was observed, with a reduction in the concentration of 5 on the first day of adulthood (Figure 4b); followed by the second day, with a reduction in the worms exposed to the highest concentrations (Figure 4c). However, on the third (Figure 4d) and fifth adult (Figure S1) days, there was no significant reduction in MV in the treated animals. The average number of MVs were also calculated over the four days (Figure S3), as the average MV area was quantified (Figure 4e), but neither result showed statistical significance.

### 3.4 ULI B Demonstrated Similar Spatial Interaction in the HRAS and LET‐60 Proteins

Molecular docking analysis was conducted using two main methods: blind and semi‐flexible docking. Blind interaction molecular docking was performed using SwisDock, which identified 34 likely interaction sites for HRAS and 36 for LET‐60 (Figure S7). This analysis demonstrated that all potential sites had similar interaction energies, ranging from −5.0 to −6.49 kcal/mol for HRAS and −5.08 to −7.65 for LET‐60. Given this ambiguity, where it was not possible to discern a primary binding site based on the energetic criterion, a more refined approach using semi‐flexible docking became necessary.

iGEMDOCK, in its semi‐flexible blind docking configuration, allows clear visualization of the likely interaction sites between ULI B and the enzymes in this study. The HRAS enzyme demonstrated an interaction energy of ‐94.23 kcal/mol, being ‐80.19 for van der Waals bonds with the amino acids ASP107, ASP108, TYR137, GLY138, GLU162, HIS166, with the respective interaction energies of −4.6, −9.4, −9.7, −11.7, −4.1, −12.5 and with interactions of −14.04 for hydrogen bonds with the amino acids ASP108, GLY138, and HIS166 and their respective interactions of −4.2, −5, −3.5. The enzyme homologous to HRAS, LET‐60, in turn, demonstrated an interaction energy of −92.1 kcal/mol, with ‐68.48 for van der Waals bonds with the amino acids ASP108, ILE139, GLU162, and ARG169, with respective interaction energies of −6.1, −7.8, −10.7, and −8.5, and interactions of −23.62 for hydrogen bonds with the amino acids GLY138, ILE139, GLU162, LYS165, and ARG169, with their respective interactions of −2.5, −3.5, −2.5, −3.5, and −9.4.

**FIGURE 5:**
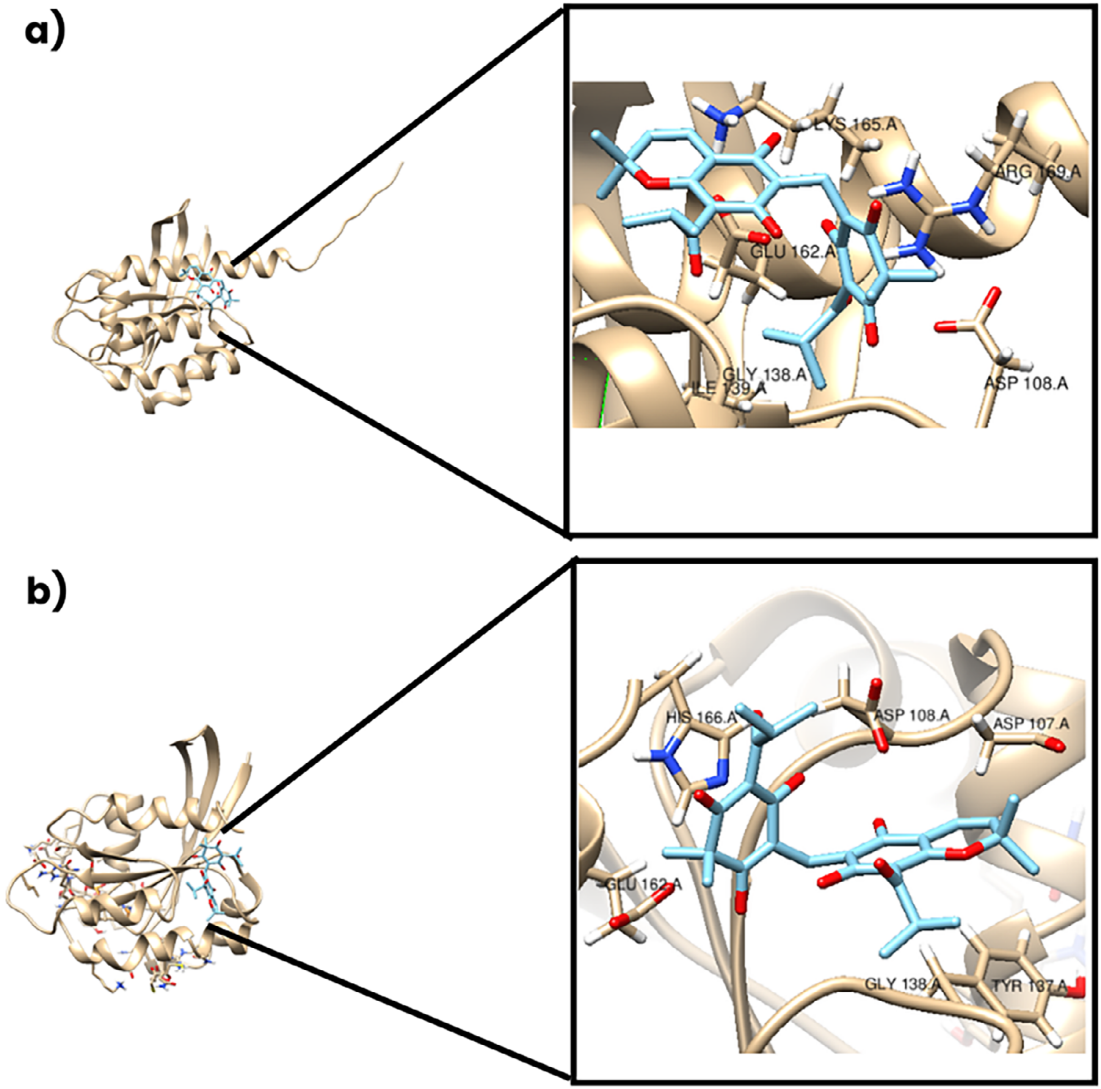
Docking molecular representation of interactions between Uliginosin B with (a) LET‐60 and (b) HRAS amino acid residues generated by iGEMDOCK.

Furthermore, the interaction regions of the bioactive compound with the enzymes were identified by SwissDock, and it was observed that the best conformation obtained by iGEMDOCK presented bonds in structurally equivalent positions, indicating that the compound occupies similar binding regions in both enzymes (human and C. *elegans*), as illustrated in Figure 5a,b.

### 3.5 ULI B Binds to an Alternative Site on LET‐60 From *C. elegans*

In this sense, to better understand the homology between proteins, we sought to analyze, through a multiple sequence alignment, the similarity and conservation of residues collected in molecular docking between EGFR from *Homo sapiens* and LET‐23 from *C. elegans*, followed by the alignment of these sequences with the folded LET‐23 protein available in the PDB. The same was performed with the GTPase HRAS isoform 1 protein from *Homo sapiens*, LET‐60 from *C. elegans*, and the folded LET‐60 protein from *C. elegans* available in Uniprot.

The alignment between the two linear sequences of the EGFR protein of *Homo sapiens* and LET‐23 of *C. elegans* showed a similarity of 32% between the sequences, and there was no conservation of any of the amino acid residues from HRAS that interacted with ULI B observed in the molecular docking (Figures S8 and S9). However, the alignment of these sequences with the folded LET‐23 protein of *C. elegans* showed similarities of 11% and 23%, indicating low conservation and only three conserved amino acid residues (VAL 899, LEU 968, and GLY 969) (Figures S10 and S11).

**FIGURE 6:**
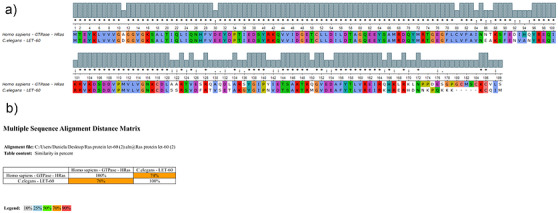
Multiple alignment of sequence. (a) Column‐to‐column map of the MUSCLE alignment of the linear amino acid sequences GTPase HRas and LET‐60. Columns show conserved regions, and below in color are the amino acids represented. (b) Alignment distance matrix of multiple sequences with the percentage representation of protein similarity found through alignment. Image generated by UGENE software.

**FIGURE 7:**
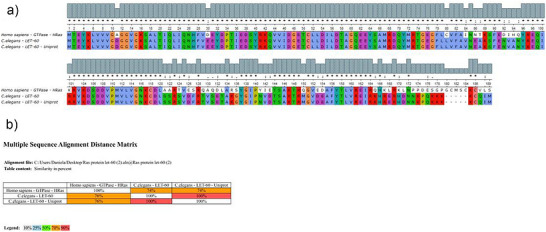
Multiple sequence alignment compared with the folded LET‐60 protein. Multiple alignment of sequence. (a) Column‐to‐column map of the MUSCLE alignment of the linear amino acid sequences GTPase HRas and LET‐60, and the folded protein of LET‐60. Columns show conserved regions, and below in color are the amino acids represented. (b) Alignment distance matrix of multiple sequences with the percentage representation of protein similarity found through alignment. Image generated by UGENE software.

The alignment between the two linear sequences of the GTPase protein HRAS isoform 1 of *Homo sapiens* and LET‐60 of *C. elegans* showed a similarity of 74% (Figure 6a,b), and the same similarity was maintained when a second alignment was performed with the folded protein of LET‐60 of *C. elegans* and these two sequences (Figure 7a,b). Only one of the residues that interacted with ULI B in the molecular docking showed conservation (LYS165). However, the percentage of similarity between these proteins indicates a high level of orthology with the human protein.

Given the high conservation of residues that interact with ULI B in molecular docking, we analyzed whether they correspond to the previously described active site of the LET‐60 protein from *C. elegans*. We annotated the regions of the previously described active site of this protein in the folded protein sequence, and then we also annotated the regions of greatest interaction of the ULI B found in molecular docking.

**FIGURE 8:**
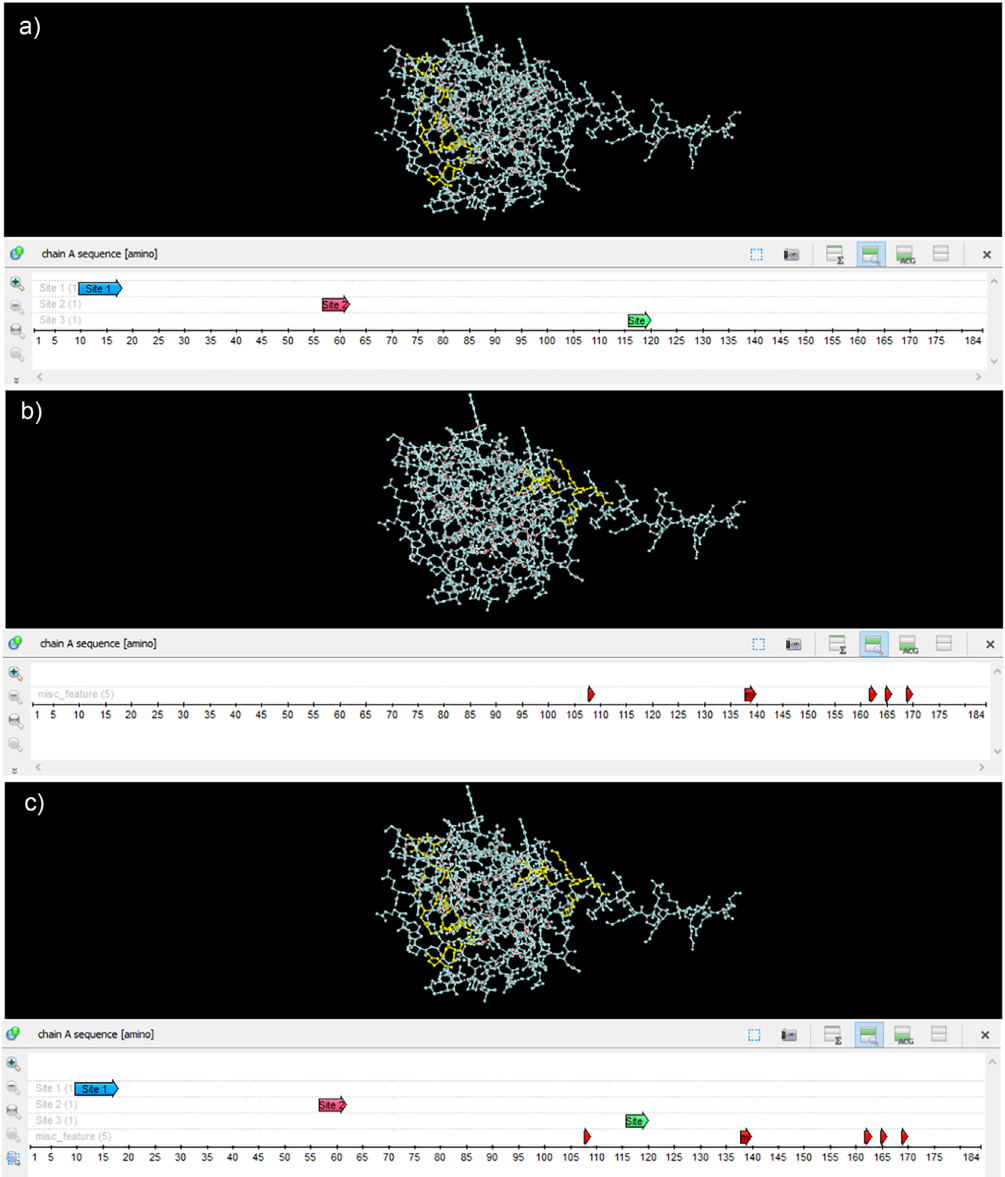
Annotation of the active sites and regions that interacted with the ULI in the LET‐60 folded protein. (a) Demonstration of the regions of the active sites on the folded protein (yellow) with its respective annotations. (b) Demonstration of the regions that interacted with the ULI in molecular docking on the folded protein (yellow) with its respective annotations. (c) Demonstration of the regions of the active sites and the regions that interacted with the ULI in molecular docking on the folded protein (yellow) with its respective annotations. Image generated by UGENE software.

Molecular docking indicated an absence of significant interaction between ULI B and the active site of LET‐60 from *C. elegans*, as defined by sites 1, 2, and 3 (Figure 8a,c). In contrast, ULI showed affinity for an alternative site, suggesting a possible allosteric mechanism (Figure 8b). All relevant regions, active site and identified alternative site, were annotated directly on the three‐dimensional structure using the UGENE software for visualization purposes (Figure 8c).

## 4 Discussion

Epidemiological data demonstrate an increase in diagnosis as well as the mortality rate resulting from cancer in the next few years [36]. Several molecules derived from natural products are already well established in cancer therapy, but there is still an increasing need for the search for drugs that present greater effectiveness and safety. Therefore, this work aimed to investigate whether ULI B would present antiproliferative effects through one of the signaling pathways already known to be involved in oncogenesis, the RAS/ERK/MAPK pathway. This work was the first in vivo study to evaluate the antiproliferative potential of ULI. It was possible to observe in *C. elegans* a small delay in the development of the multi‐vulva phenotype caused by increased Ras pathway signaling. However, these results were not as potent as we had hypothesized. These findings were corroborated by molecular docking analysis, which demonstrated that ULI B interacts with LET‐60 at alternative sites of the protein through an allosteric modulation (Figures 5 and 8). This was different from the interactions with the human protein, which occurred with specific sites of the human active and inactive protein models studied (Figure S6; Table S1 and Table S3). These data indicate that the worm protein is structurally different from the human and that ULI B may present a more powerful effect in human cancer models.

As demonstrated in both survival and body size assays, no significant changes in relation to control were observed, therefore, ULI B proved to be safe for the MT4244 strain at the concentrations tested. Notably, in previous analyses performed in our laboratory, the N2 strain exposed to ULI B exhibited significant mortality at 20 µM, with an estimated LC50 of 14.23 µM [33]. Therefore, we tested lower concentrations in this study. In other cases, such as the evaluation of the LaSOM 335 molecule in N2 and MT4244 strains, a reduction in survival was observed in both strains, starting at a concentration of 600 µM [37]. This was also the case in the evaluation of the drugs itraconazole, disulfiram, ouabain, and etodolac, which also showed mortality only at higher concentrations (600 and 1000 µg/mL, approximately 850–2000 µM) [30]. Thus, ULI B causes cytotoxicity and antiproliferative effects at lower concentrations.

**FIGURE 9:**
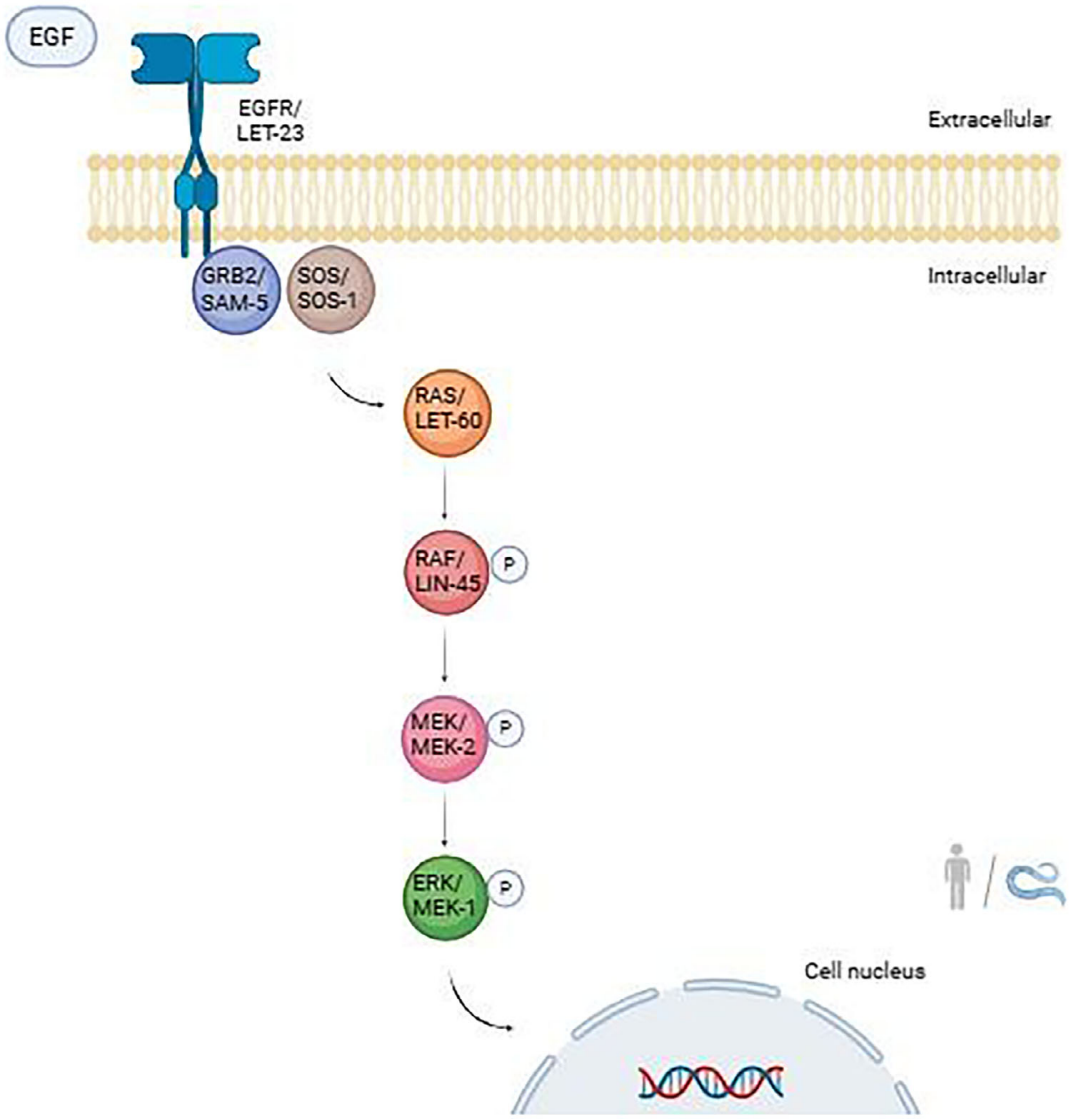
RAS signaling pathway. Comparison between human proteins and homologous nematode proteins. EGF: endothelial growth factor; EGFR: endothelial growth factor receptor; GRB2: Growth factor receptor‐bound protein 2; SOS: son of sevenless; RAS: rat sarcoma virus; RAF: protein‐serine/threonine kinase; MEK: mitogen‐activated protein kinase; ERK: extracellular signal‐regulated kinase. Adapted from Ávila and Gubert (2022).

Then, we assessed the antiproliferative effect of ULI B in *C. elegans*. ULI B delayed the formation of the multivulva phenotype in strain MT4244; however, as the days passed by, it did not prevent the formation of tumors. We hypothesize that this was due to the short‐term exposure to ULI, which lasted only 30 min, in order to avoid its toxicity, it was not sufficient to obtain a more potent action in reducing MV, being one of the limitations of our study. This strain is understood to be related to overstimulation of the RAS pathway, which is highly conserved in the human RAS signaling pathway. The pathway is activated through the transmembrane receptor EGFR (epidermal growth factor receptor), orthologous to LET‐23 in *C. elegans*, which ligands are endothelial growth factors (EGR), transforming growth factors (TGF), or tumor necrosis factor. This pathway begins with the interaction of the intracellular Grb2/SOS complex, which will couple to the receptor and activate the Ras protein (G protein), followed by a cascade of phosphorylation and activation of the intracellular proteins Raf kinase, MEK (MAP kinase‐kinase), and ERK MAPK (MAP kinase) (Figure 9). ERK MAPK regulates target genes in the cell nucleus, responsible for cell growth, proliferation, differentiation and apoptosis [23, 25]. When this pathway is altered, tumorigenesis can occur. Some examples are mutations in the *ras* gene, which is present in around 30% of all types of cancer, in addition to receptor tyrosine kinases (RTKs), whose increased expression or activation by its ligands leads to the process of angiogenesis and cancer progression, and may even lead to the development of metastasis [24, 26]. To investigate possible mechanisms of action of ULI B, additional assays were performed; however, none of them yielded significant results (Figure S2).

Previously, a study investigated the antitumor effects of itraconazole, disulfiram, etodolac and ouabain, using different types of tumorous *C. elegans* strains, including those associated with the Ras pathway (SD939 and MT2124). Remarkably, they all demonstrated the potential to reduce the multivulva phenotype in the MT2124 strain at concentrations between 370 and 400 µg/mL [30]. Our group has investigated LASOM 335, a dihydropyrimidinone derivative, which demonstrated promising activity in reducing the phenotype of the transgenic strain MT4244 [37]. Similar effects have already been observed using the molecule harmine, which was able to reduce the percentage of animals with MV phenotype in transgenic strains with overexpression of LET‐23 with mutations of the tyrosine kinase T790M–L858R. Furthermore, this study stands out by reducing the MV effect in transgenic strains with hyperactivation of *let‐60* and *lin‐45* (ortholog of the human B‐Raf proto‐oncogene) at concentrations between 5 and 160 µM of harmine [38]. Therefore, the intermediates of the RAS pathway are also targets of interest for the activity of molecules with anticarcinogenic action.

Finally, we sought to understand the interaction of ULI B with the target protein LET‐23 in *C. elegans*, as well as its human ortholog EGFR, using in silico assays, such as molecular docking. Docking is an approach that uses software to identify, optimize, and reposition drugs, detecting targets by reverse screening, and helping to understand the relationships between different molecular targets for a given disease [39]. Muscle is a computational method that uses software algorithms to rapidly generate high‐accuracy multiple alignments of protein sequences, optimize the alignment through iterative refinement, reduce computational costs by estimating sequence distances efficiently, and improve overall alignment quality for comparative and evolutionary analyses in bioinformatics [40]. In this work, it was chosen to evaluate the interaction of ULI B in the tyrosine kinase domain of EGFR and the GTPase protein HRAS, both the human structure and that of *C. elegans*, due to its availability in the PDB and Uniprot, named LET‐23 and LET‐60, respectively.

EGFR dimerizes after ligand binding to its extracellular domain, activating a signaling cascade. Mutations in its tyrosine kinase domain are associated with alterations in cell proliferation, angiogenesis, and metastasis, making it a target for chemotherapeutic agents [41]. Our results show that the C797 residue of the human EGFR model is interacting covalently with ULI B, paramount for its efficacy and inhibition of variants containing C797S.[21] In addition to the interaction with the amino acids LEU 718, PHE 723, and ASP 800, other promising molecules tested also interact (Table S3; Figure S6) [42]. In LET‐23, ULI B did not present similar bindings as the main ligand ATP (Table S1), which may explain its lower effect in preventing the formation of the MV phenotype in the model used in this study (Table S2 and Figure S5) [43]. An interaction test was also performed on the inactive conformation of EGFR, as in previous studies, but which did not demonstrate critical interactions for inhibitory action (Table S1) [44, 45].

**FIGURE 10:**
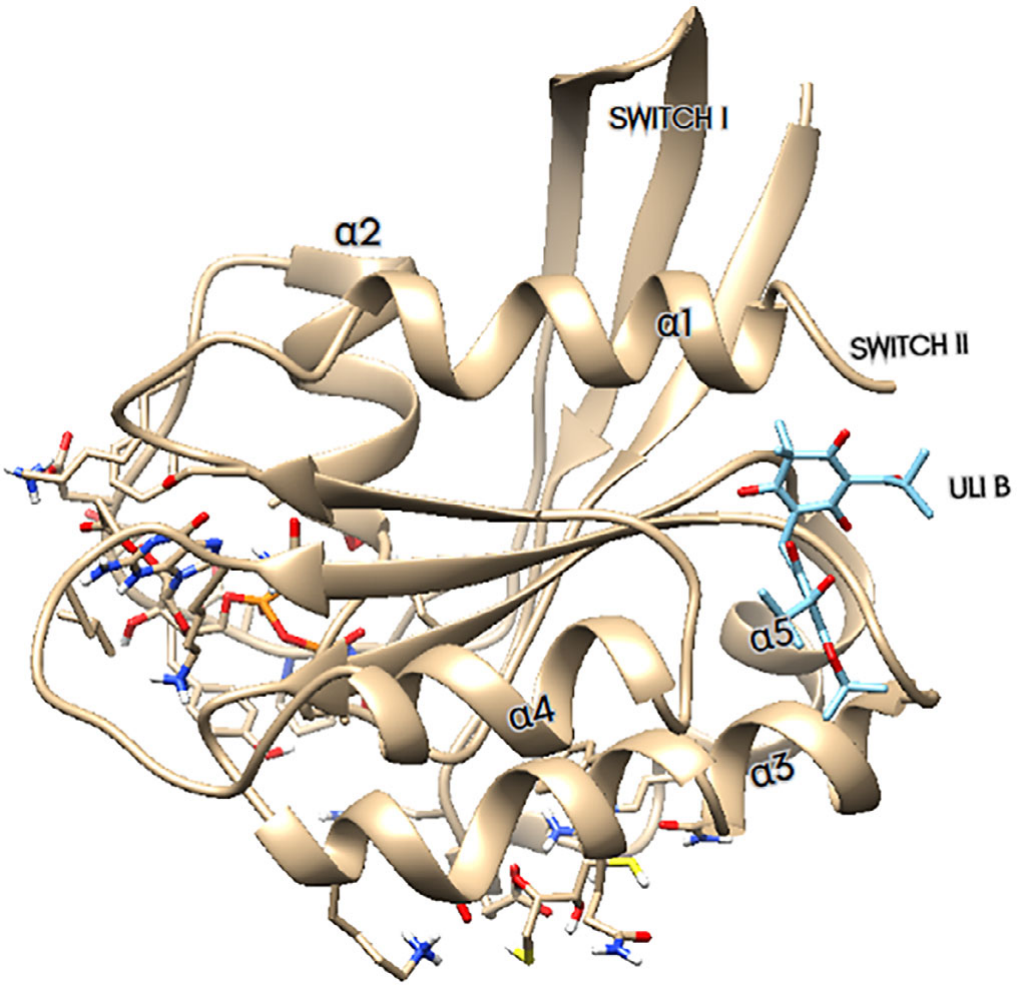
Structural representation of the HRAS, highlighting the main structural regions and the location of ULI B (created by the authors).

The allosteric lobe of the RAS protein, located in the α4 and α5 helices, plays a fundamental role in the autoregulation and dimerization of the protein, critical steps for the activation of RAS‐mediated signaling. Previous studies have shown that mutations in this region do not abolish the self‐association capacity of RAS. However, it has been investigated that the binding of the NS1 monobody to the allosteric lobe promotes the disorganization of RAS clusters in the membrane, interrupting signaling by preventing dimer formation and reducing interaction with effectors, such as the RAF protein [46]. Although the allosteric lobe of HRAS remains underexplored computationally, studies of the paralogous protein KRAS already characterize this region as a promising therapeutic target. Wang et al. mapped the main binding sites of KRAS, identifying pockets S1, S2, and S3, the latter being associated with the α5 helix and the allosteric lobe [47]. Given this classification, the docking results indicate that ULI B interacts predominantly with the allosteric lobe of HRAS. ARG169, GLY138, and ILE139 residues are located in pocket S3; LYS165, and GLU162 are located in pocket S2, between helices α3 and α4; while ASP108 is found in the region between Switch II and helix α3 (Figure 10). This interaction pattern supports the hypothesis of an allosteric mechanism of action for ULI B.

However, a study that determined and analyzed the sequence of the EGFR domains with that of *C. elegans*, the LET‐23 homologous receptor, indicated that it does not share a part of the canonical sequences, which can alter its allosteric activation compared to the human receptor [48]. This can corroborate with the differences in affinity and interactions identified in our results, which showed greater specificity of ULI B with human proteins (7KXZ and 1M14) compared to that of *C. elegans* (5WNO) (Figure S4), in addition to the alignment of the human and *C. elegans* models (7KXZ and 5WNO). Multiple sequence alignments were used to investigate the homology and conservation of residues involved in interaction with the ULI B ligand across human proteins and their *C. elegans homologs*. Comparative sequence and structural analyses revealed significant differences in the degree of conservation between human proteins and their homologs, affecting the interpretation of molecular docking results. To determine probable structural and functional conservation between these proteins, the percentage of similarity obtained through the alignment of these sequences was used as a reference, where values greater than 40% indicate a high homology between them [49, 50, 51].

EGRF and LET‐23 showed low similarity between linear sequences (32%) and a lack of conservation of residues that interacted with ULI B in molecular docking. When aligned with the folded structure of LET‐23, the percentage of similarity was even lower (11%–23%), with only three conserved residues, also demonstrating low structural and functional conservation of the interaction sites with ULI B. In contrast, the alignment between the linear sequences of human HRAS and *C. elegans* LET‐60 showed high similarity (74%), thus maintaining alignment with the folded structure of LET‐60. However, despite the high conservation, the residues that interacted with ULI B were not conserved, except for a single amino acid (LYS 165), suggesting that the ligand–protein interaction does not occur in canonically conserved regions.

Structural analysis showed that ULI B does not interact directly with any of the three active‐site regions of *C. elegans* LET‐60 (human RAS) that have already been described in the literature. Instead, we observed that the ligand showed affinity for an alternative, spatially distinct site, suggesting a possible allosteric modulation mechanism. In this sense, this binding of ULI B to an alternative active site may help us understand why ULI B is able to slightly reduce the growth of multi‐vulvae in the MT4244 strain without completely inhibiting their growth.

Our in‐silico analyses suggested that the interaction of ULI B with LET‐60 may present an allosteric modulation mechanism, since the binding occurs at an alternative site to the protein active site. The *in vivo* assays showed that exposure was not able to inhibit the proliferation of the multi‐vulva phenotype caused by LET‐60 overexpression, but it did significantly delay its manifestation. This partial effect may indicate compatibility with a non‐competitive mechanism, where binding to an allosteric site may result in modulation of protein activity without causing complete inhibition. Furthermore, LET‐60 overexpression in the MT4244 strain may contribute to the maintenance of the phenotype, since even under conditions of exposure to ULI B, the elevated protein levels could overcome the modulation exerted by the compound.

Therefore, this study is justified as a search for new tyrosine kinase and RAS inhibitors, since around 70% of the drugs already developed cause some degree of hepatotoxicity, and some have even been withdrawn from the market [52]. It is noteworthy that research into the RAS signaling pathway, both in the in‐silico model and the in vivo model in *C. elegans*, is still scarce in the literature, highlighting the importance of these findings. However, it is necessary to continue to elucidate the antiproliferative mechanisms of ULI B and other promising molecules at other EGFR activation sites, as well as in other proteins involved in the RAS pathway. ULI B demonstrated an affinity for interaction with human EGFR, therefore the investigation of its antiproliferative effect in other in vivo models, as in other proteins of the RAS pathway, is still necessary.

## 5 Conclusion

According to the results found in this study, a potential delay in the development of the mv MV phenotype in the strain MT4244 was observed in the first two days of adulthood. We sought to understand the possible mechanism of this phenomenon by investigating its interaction with human HRAS and nematode LET‐60 proteins. The molecular docking complemented in vivo findings, using the *C. elegans* model, and the multiple sequence alignments revealed a high specificity with the LET‐60 from *C. elegans*, which also showed high similarity and homology with the HRAS from humans. These analyses indicated that ULI B interacts with LET‐60 through a possible allosteric and non‐competitive modulation mechanism, resulting in a partial effect characterized by delaying, without inhibiting, the multi‐vulva phenotype. This corroborates the results observed in in vivo assays. Furthermore, the application of the flexible redocking method confirmed a possible interaction with the tyrosine kinase domain of this receptor, suggesting a potential antiproliferative effect in human cells.

## Author Contributions

**Gilsane von Poser**: conceptualization, supervision, resources, writing – review and editing. **Flávia Suelen de O. Pereira**: investigation, writing – review and editing, methodology. **Helder Dias Costa**: investigation, writing – original draft, methodology, data curation. **Favero R. Paula**: conceptualization, writing – review and editing, resources, supervision. **Tarsila Dantas da Rosa**: investigation, methodology. **Maria Eduarda O. de Souza**: conceptualization, investigation, writing – original draft, methodology, formal analysis. **Daniela Teixeira Rodrigues**: investigation, writing – original draft, methodology, data curation. **Stela M. K. Rates**: conceptualization, writing – review and editing, supervision, resources. **Daiana Silva Ávila**: conceptualization, writing – review and editing, supervision, resources, project administration. **Camila Machado Pires da Silva**: investigation, methodology.

## Conflicts of Interest

The authors declare no conflicts of interest.

## Supporting information




**Supporting File 1**: cbdv71592‐sup‐0001‐SuppMat.docx.

## Data Availability

The data that support the findings of this study are available on request from the corresponding author. The data are not publicly available due to privacy or ethical restrictions.
