# Supplementary material for: Evaluation of Uliginosin B as a Modulator of the Ras Pathway: In Vivo Evidence From Caenorhabditis elegans and In Silico Insights
Source: Chem Biodivers. 2026 Aug 17;23(8):e71592. doi: 10.1002/cbdv.71592 (PMC13480929; doi:10.1002/cbdv.71592)
Supplement: Supplementary file 1 [file CBDV-23-e71592-s001.docx]

Supplementary Material

**FIGURE S1** - Analysis of the multivulva (MV) phenotype on the 5th day of adulthood of the worms (n=4). Data were parametric and ANOVA was performed, followed by Dunnett’s multiple comparison test. Data expressed as mean ± SEM. (*) Indicates a statistically significant difference in relation to the control group with **p < 0.05 **p < 0.01 ***p<0.001 ****p < 0.0001.*

**FIGURE S2** - Mechanisms assays. a) Analysis of apoptotic bodies in the germline in strain MD701 (n=4). b) Quantification of lipofuscin fluorescence in N2 and MT4244 strains (n=4). Data were parametric and ANOVA was performed, followed by Dunnett’s multiple comparison test. Data were normalized to 100% of control and expressed as mean ± SEM. (*) Indicates a statistically significant difference in relation to the control group with **p < 0.05 **p < 0.01 ***p<0.001 ****p < 0.0001.*

**FIGURE S3** - Quantification of the average number of MV in the MT4244 strain. a) On the first day, b) second, c) third, d) and fifth day of adult worm (n=4). (a) Data were non-parametric and Kruskal-Wallis followed by Dunn's multiple comparisons test was applied. (b, c and d) Data were parametric and ANOVA was performed, followed by Dunnett’s multiple comparison test. Data expressed as mean ± SEM. (*) Indicates a statistically significant difference in relation to the control group with **p < 0.05 **p < 0.01 ***p<0.001 ****p < 0.0001.*

**TABLE S1** - Total interaction energy, Van der Waals and hydrogen bonds of the interaction with ULI B in the inactive conformation of tyrosine kinase domain of EGFR (1M14), and the main amino acids.

**FIGURE S4** – Blind molecular docking with target proteins. (a) Possible sites of interaction of ULI B with the human protein in the active form 7KXZ. (b) Interactions of ULI B with the *C. elegans* 5WNO protein. (c) Interactions of ULI B with the human protein in the inactive form 1M14.

a)

b)	c)

**FIGURE S5** - a) Illustration of the structure of the LET-23 tyrosine domain and binding to the ULI B and ATP molecules. b) and c) Interaction of the ULI B and the ATP molecule in the LET-23 tyrosine kinase domain. Demonstration of the amino acids that interacted with the ULI B.

**TABLE S2** - Total interaction energy, van der Waals and hydrogen bonds of the ULI B in the tyrosine kinase domain of LET-23, and main amino acids.

a)

b)	c)

**FIGURE S6** - a) Illustration of the structure of the EGFR tyrosine domain and superimposed ULI B and BI-2040 molecules. b) and c) Interaction of ULI B and the BI-2040 molecule in the EGFR tyrosine kinase domain. Demonstration of the amino acids that interacted with ULI B.

**TABLE S3** - Total interaction energy, Van der Waals and hydrogen bonds of the interaction with ULI B in the tyrosine kinase domain of EGFR, and the main amino acids.

**FIGURE S7** – Blind molecular docking with target proteins. (a) Possible sites of interaction of ULI B with the human HRAS protein. (b) Interactions of ULI B with the *C. elegans* LET-60 protein.

**FIGURE S8** – Multiple alignment of sequence. Column-to-column map of the MUSCLE alignment of the linear amino acid sequences EGFR protein and LET-23. Columns show conserved regions, and below in color are the amino acids represented. Image generated by UGENE software.

**FIGURE S9** – Alignment distance matrix of multiple sequences with the percentage representation of protein similarity found through alignment. Image generated by UGENE software.

**FIGURE S10** – Multiple alignment of sequence. Column-to-column map of the MUSCLE alignment of the linear amino acid sequences EGFR protein and LET-23, and the folded protein of LET-23. Columns show conserved regions, and below in color are the amino acids represented. Image generated by UGENE software.

**FIGURE S11** – Alignment distance matrix of multiple sequences with the percentage representation of protein similarity found through alignment. Image generated by UGENE software.
